# The association of body mass index with long-term clinical outcomes after ticagrelor monotherapy following abbreviated dual antiplatelet therapy in patients undergoing percutaneous coronary intervention: a prespecified sub-analysis of the GLOBAL LEADERS Trial

**DOI:** 10.1007/s00392-020-01604-1

**Published:** 2020-01-31

**Authors:** Masafumi Ono, Ply Chichareon, Mariusz Tomaniak, Hideyuki Kawashima, Kuniaki Takahashi, Norihiro Kogame, Rodrigo Modolo, Hironori Hara, Chao Gao, Rutao Wang, Simon Walsh, Harry Suryapranata, Pedro Canas da Silva, James Cotton, René Koning, Ibrahim Akin, Benno J. W. M. Rensing, Scot Garg, Joanna J. Wykrzykowska, Jan J. Piek, Peter Jüni, Christian Hamm, Philippe Gabriel Steg, Marco Valgimigli, Stephan Windecker, Robert F. Storey, Yoshinobu Onuma, Pascal Vranckx, Patrick W. Serruys

**Affiliations:** 1 Amsterdam UMC, Heart Center, Department of Clinical and Experimental Cardiology, Amsterdam Cardiovascular Sciences, University of Amsterdam, Meibergdreef 9, Amsterdam, The Netherlands; 2 Division of Cardiology, Department of Internal Medicine, Faculty of Medicine, Prince of Songkla University, Songkhla, Thailand; 3 Erasmus Medical Centre, Thoraxcentre, Rotterdam, the Netherlands; 4 First Department of Cardiology, Medical University of Warsaw, Warsaw, Poland; 5 Department of Internal Medicine, Cardiology Division, University of Campinas (UNICAMP), Campinas, Brazil; 6 Department of Cardiology, Radboud University Medical Center, Nijmegen, The Netherlands; 7 Depatment of Cardiology, Xijing hospital, Xi’an, China; 8 Belfast Health and Social Care Trust, Cardiology, Belfast, Ireland; 9 Serviço de Cardiologia, Hospital de Santa Maria, Lisbon, Portugal; 10 Department of Cardiology, Heart and Lung Centre, New Cross Hospital, Wolverhampton, UK; 11 Cardiology Service, Saint Hilaire Clinic, Rouen, France; 12 First Department of Medicine, University Medical Centre Mannheim (UMM), Faculty of Medicine Mannheim, University of Heidelberg, Mannheim, Germany; 13 Department of Cardiology, St. Antonius Hospital, Nieuwegein, The Netherlands; 14 Department of Cardiology, Royal Blackburn Hospital, Blackburn, UK; 15 Applied Health Research Centre, Li Ka Shing Knowledge Institute, St Michael’s Hospital, University of Toronto, Toronto, Canada; 16 University of Giessen and Kerckhoff Heartand Thorax Center, University of Giessen, Bad Nauheim, Germany; 17 FACT (French Alliance for Cardiovascular Trials), Université de Paris, Assistance Publique-Hôpitaux de Paris -Diderot, Paris, France; 18 Department of Cardiology, University of Bern, Inselspital, Bern, Switzerland; 19 Cardiovascular Research Unit, Department of Infection, Immunity and Cardiovascular Disease, University of Sheffield, Sheffield, UK; 20 Department of Cardiology, NUIG (National University of Ireland, University Road, Galway)Galway, H91 TK33 Ireland; 21 Jessa Ziekenhuis, Faculty of Medicine and Life Sciences at the Hasselt University, Hasselt, Belgium; 22 NHLI, Imperial College London, London, UK

**Keywords:** Body mass index, Percutaneous coronary intervention, Drug-eluting stent, Dual antiplatelet therapy, Ticagrelor monotherapy, Acute coronary syndrome

## Abstract

**Background:**

The efficacy of antiplatelet therapies following percutaneous coronary intervention (PCI) may be affected by body mass index (BMI).

**Methods and results:**

This is a prespecified subgroup analysis of the GLOBAL LEADERS trial, a prospective, multicenter, open-label, randomized controlled trial in an all-comer population undergoing PCI, comparing the experimental strategy (23-month ticagrelor monotherapy following 1-month dual antiplatelet therapy [DAPT]) with a reference regimen (12-month aspirin monotherapy following 12-month DAPT). A total of 15,968 patients were stratified by baseline BMI with prespecified threshold of 27 kg/m^2^. Of those, 6973 (43.7%) patients with a BMI < 27 kg/m^2^ had a higher risk of all-cause mortality at 2 years than those with BMI ≥ 27 kg/m^2^ (adjusted HR 1.24, 95% CI 1.02–1.49). At 2 years, the rates of the primary endpoint (all-cause mortality or new Q-wave myocardial infarction) were similar between treatment strategies in either BMI group (*p*_interaction_ = 0.51). In acute coronary syndrome, however, the experimental strategy was associated with significant reduction of the primary endpoint compared to the reference strategy in patients with BMI < 27 kg/m^2^ (HR 0.69, 95% CI 0.51–0.94), but not in the ones with BMI ≥ 27 kg/m^2^ (*p*_interaction_ = 0.047). In chronic coronary syndrome, there was no between-group difference in the efficacy and safety of the two antiplatelet strategies.

**Conclusions:**

Overall, BMI did not influence the treatment effect seen with ticagrelor monotherapy; however, a beneficial effect of ticagrelor monotherapy was seen in ACS patients with BMI < 27 kg/m^2^.

**Trial registration:**

The trial has been registered with ClinicalTrials.gov, Number NCT01813435.

**Graphic abstract:**

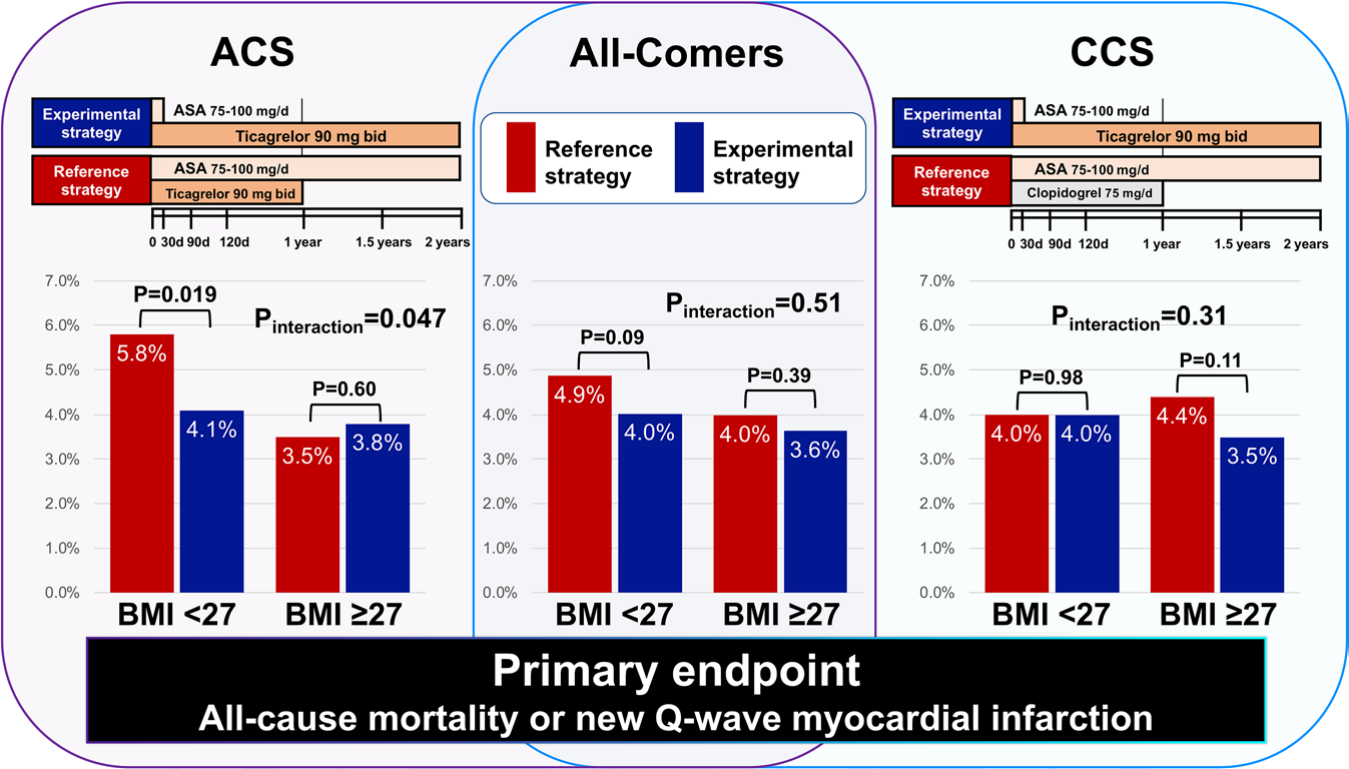

**Electronic supplementary material:**

The online version of this article (10.1007/s00392-020-01604-1) contains supplementary material, which is available to authorized users.

## Introduction

Body mass index (BMI) is simple to calculate and consequently used as an indicator of general adiposity [1]. Although obesity is well recognized as a major risk factor of cardiovascular disease (CVD) [2], numerous studies have demonstrated a paradoxical association between higher BMI and lower risk of adverse events in patients with established CVD, even after adjusting for confounding factors. In this phenomenon, dubbed the “obesity paradox” [3–5], patients with lower or even normal BMI have a higher risk of both ischemic and bleeding events after percutaneous coronary intervention (PCI) compared to those who are overweight [6]. To date, however, no tailored antiplatelet strategy has been recommended for these patients [7].

It is recognized that the efficacy of platelet inhibition due to antiplatelet therapy including novel potent P2Y12 inhibitors could be associated with a patient’s BMI [8]. In other words, high or low BMI could lead to an inappropriate balance between anti-ischemic and bleeding risks [9–11]. Therefore, assessment of different antiplatelet strategies after PCI, stratified according to BMI, may provide additional insight into patients with a “high-risk” BMI.

The GLOBAL LEADERS trial compared the experimental antiplatelet regimen with 23-month ticagrelor monotherapy, to the reference regimen of conventional 12-month dual antiplatelet therapy (DAPT) followed by 12-month aspirin in an all-comers PCI population [12]. The superiority of the experimental strategy at 2 years was not demonstrated in the parent trial. However, non-specified secondary analyses suggested the potential efficacy of this novel experimental regimen in some specific patient subgroups [12–15]. To unravel the complex intricacies of the GLOBAL LEADERS trial, the present study aims to investigate the clinical impact of BMI on the novel antiplatelet strategy with ticagrelor monotherapy in patients undergoing PCI.

## Methods

### Study design

This study is a prespecified subgroup analysis of the GLOBAL LEADERS trial [16]. The GLOBAL LEADERS trial [12] is a multi-center, prospective, open-label randomized controlled trial in an all-comer population with no restriction regarding clinical presentation, complexity of the lesions or number of stents used (NCT01813435). Details of the study design and protocol have been reported elsewhere [16]. In brief, the trial randomly assigned patients before PCI to either (i) the experimental strategy with 1-month DAPT (aspirin 75–100 mg daily and ticagrelor 90 mg twice daily) followed by 23-month ticagrelor 90 mg twice daily monotherapy, or (ii) the reference regimen with 12-month DAPT [aspirin 75–100 mg daily and either ticagrelor 90 mg twice daily for acute coronary syndromes (ACS: unstable angina, non ST-elevation myocardial infarction, and ST elevation myocardial infarction) or clopidogrel 75 mg daily for chronic coronary syndromes (CCS)] followed by 12-month aspirin 75–100 mg daily monotherapy, respectively. All target lesions were treated by default with biolimus A9-eluting stents (BioMatrix, Biosensors, Europe). The trial was approved by the institutional review board at each center and followed the ethical principles of the Declaration of Helsinki. All the patients gave written informed consent prior to participation in the trial.

### Patients population and study endpoints

The patient’s baseline BMI was calculated as weight in kilograms divided by height in meters squared collected at the time of randomization. Patients were divided into two groups according to a threshold BMI of 27.0 kg/m^2^, which was prespecified in the design paper [16] and adopted by reference to previous publications [17, 18], and also corresponds to the median value of BMI in the present population. In each BMI group, clinical, demographic, angiographic, and procedural characteristics were compared between patients who received the experimental and reference antiplatelet regimen.

The primary endpoint of this study was the composite of all-cause mortality and new Q-wave myocardial infarction (MI) up to 2 years. Deaths from any cause were ascertained without the need for adjudication [19, 20]. Q-wave MI was centrally adjudicated by an independent electrocardiogram core lab and defined in accordance with the Minnesota classification (new major Q–QS wave abnormalities) or by the appearance of a new left bundle branch block in conjunction with abnormal biomarkers [21]. The secondary safety endpoint was major bleeding events according to the Bleeding Academic Research Consortium (BARC) criteria type 3 or 5 [22]. Additional endpoints included stroke (ischemic or hemorrhagic), BARC type 2 bleeding, definite stent thrombosis according to Academic Research Consortium (ARC) definition [23], and the composite of all-cause mortality, any stroke, and new Q-wave MI [16]. The composite endpoints were analyzed according to time-to-first event analysis.

### Statistical analysis

Continuous variables are reported as mean ± standard deviations (SD) or median and interquartile range (IQR), and are compared using Student’s *t* tests or Mann–Whitney *U* test, respectively. Categorical variables are reported as percentages and numbers and are compared using Chi-square or Fisher’s exact test as appropriate.

Association between baseline BMI as a continuous variable and adverse outcomes including the primary and secondary endpoint is depicted using restricted cubic spline function from the adjusted Cox regression model. Kaplan–Meier method is used to estimate the cumulative rates of clinical events and log-rank test is performed to examine the differences between groups. The effect of BMI on the outcomes is assessed in the unadjusted and adjusted Cox proportional hazards model. The clinical outcomes were compared stratified according to both the prespecified threshold of 27 kg/m^2^ and the World Health Organization (WHO) classification**:** underweight (BMI < 18.5 kg/m^2^), normal weight (BMI 18.5–24.9 kg/m^2^), overweight (BMI 25.0–29.9 kg/m^2^), and obesity (BMI ≥ 30 kg/m^2^). The covariables in the adjusted model are listed in Fig. 2 and Table 2**,** which were selected based on previous knowledge and literature [24, 25]. Variance inflation factor (VIF) of covariables are calculated to confirm the absence of multicollinearity. We also performed the receiver operating characteristic (ROC) analysis to detect the optimal cutoff value of BMI for predicting the primary endpoint according to the Youden index. The treatment effect of the experimental vs. the reference strategy between subgroups is estimated with an unadjusted Cox regression model.

Because different P2Y_12_ inhibitors in the reference group were used depending on clinical presentation (ticagrelor for ACS or clopidogrel for CCS), the prespecified stratified analysis according to clinical presentation is performed. In addition, landmark analyses are reported using the prespecified time points of 1 year (at the time of the planned cessation of a P2Y_12_ inhibitor in the reference strategy).

Statistical significance was considered if two-sided *p* value was less than or equal 0.05. All analyses were performed in SPSS Statistics, version 26 (IBM Corp., Armonk, 281 N.Y., USA) and R software version 3.5.1 (R Foundation for Statistical Computing, Vienna, Austria).

## Results

**Fig. 1:**
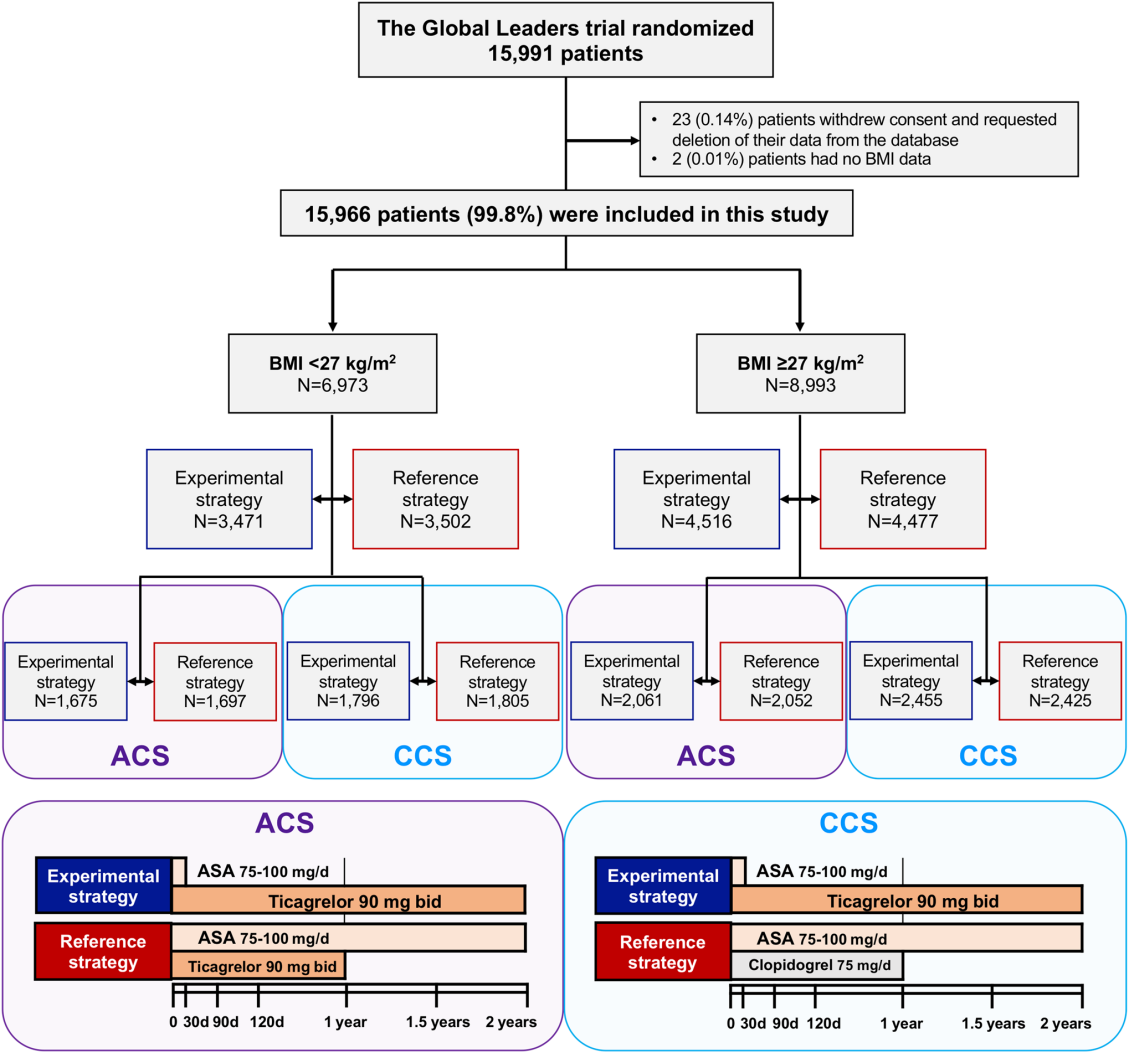
Flowchart of the present study. Among 15,966 patients included in this analysis, 6973 (43.7%) had BMI < 27 kg/m^2^ and 8993 patients (56.3%) had BMI ≥ 27 kg/m^2^. Outcomes were assessed between experimental strategy and reference strategy in all-comers population, and furthermore in each clinical presentation (ACS and CCS). *BMI* body mass index, *ACS* acute coronary syndrome, *CCS* chronic coronary syndrome, *ASA* acetylsalicylic acid

**Fig. 2:**
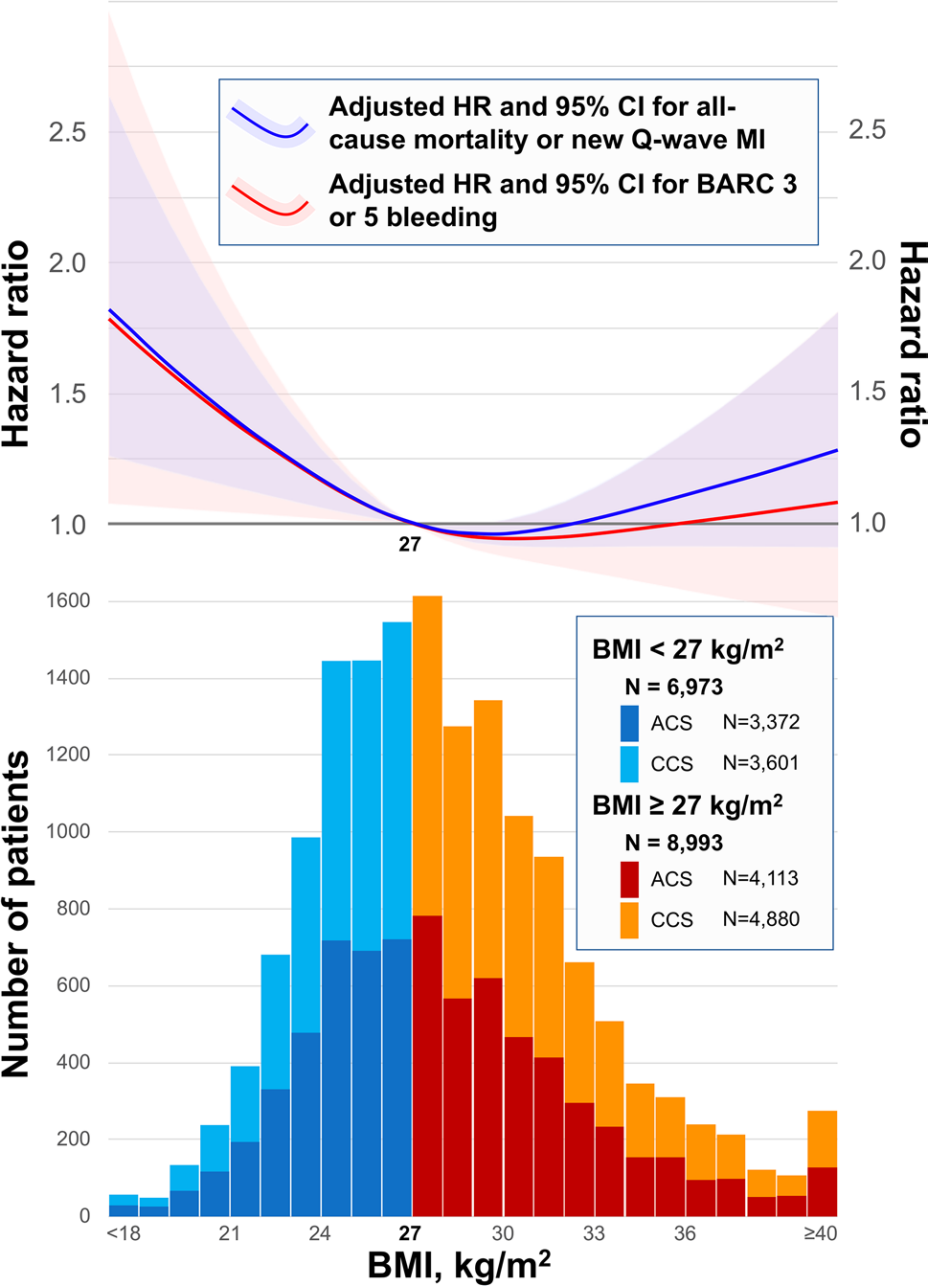
Histogram of BMI stratified by clinical presentation with adjusted hazard ratio for adverse events according to BMI. Blue and red bar graphs indicate the number of patients with BMI < 27 kg/m^2^ and ≥ 27 kg/m^2^ in the setting of ACS, respectively. Similarly, sky blue and orange bar graphs indicate the number of patients with BMI < 27 kg/m^2^ and ≥ 27 kg/m^2^ in the setting of CCS, respectively. Blue curve with light blue area indicates adjusted hazard ratio with 95% CI for composite of all-cause mortality and new Q-wave MI at 2-year according to BMI with reference of 27 kg/m^2^. Red curve with light red area indicates adjusted hazard ratio with 95% CI for BARC type 3 or 5 bleeding according to BMI with reference of 27 kg/m^2^. The number of knots for the cubic spline curves were three in each model. Adjusted covariates for all-cause mortality or new Q-wave MI are age (years), sex, clinical presentation (ACS or CCS), diabetes mellitus, hypertension, hypercholesteremia, PVD, COPD, renal impairment, previous MI, previous PCI, and previous CABG. Adjusted covariates for BARC type 3 or 5 bleeding are age (years), sex, clinical presentation (ACS or CCS), diabetes mellitus, previous bleeding, renal impairment, anemia according to WHO classification, and radial access in the index procedure. *BMI* body mass index, *ACS* acute coronary syndromes, *CCS* chronic coronary syndromes, *HR* hazard ratio, *CI* confidence interval, *MI* myocardial infarction, *BARC* Bleeding Academic Research Consortium, *PVD* peripheral vascular disease, *COPD* chronic obstructive pulmonary disease, *PCI* percutaneous coronary intervention, *CABG* coronary artery bypass graft, *WHO* World Health Organization

A total of 15,991 patients at 130 hospitals in 18 countries were enrolled in the GLOBAL LEADERS trial between 1st July 2013 and 9th November 2015; of these 23 patients withdrew their consent and their data were deleted from the database. Of the remaining 15,968 patients included in the main study, baseline BMI was available in 15,966 patients (99.99%) (Fig. 1). The median BMI was 27.68 (interquartile range 25.00–30.69) kg/m^2^ with 6973 (43.7%) patients with a BMI < 27 kg/m^2^ and 8993 (56.3%) patients with a BMI ≥ 27 kg /m^2^. The distribution of patients according to BMI is shown in Fig. 2.

### Baseline characteristics

A comparison of the baseline characteristics between the two BMI groups is shown in Table 1. Compared to those with a BMI ≥ 27 kg/m^2^, patients with BMI < 27 kg/m^2^ were older; more likely to present with ACS; had lower prevalence of diabetes, hypertension, hypercholesterolemia, renal impairment, previous MI, and previous PCI; were more likely to be smokers, and had a higher prevalence of peripheral artery disease. Patients with a BMI < 27 kg/m^2^ had higher rates of PCI in the left anterior descending artery compared to those with a BMI ≥ 27 kg/m^2^. There were no significant differences in the rates of radial access between the two BMI groups.

**Table 1:** Comparison of clinical and angiographic characteristics between patients with BMI < 27 kg/m^2^ and ≥ 27 kg/m^2^

	BMI < 27 kg/m^2^	BMI ≥ 27 kg/m^2^	*p* value
	*N* = 6973/15,966 (43.7%)	*N* = 8993/15,996 (56.3%)	
Age (years)	65.6 ± 10.5	63.7 ± 10.1	< 0.001
BMI (kg/m^2^)	24.3 ± 2.0	31.2 ± 3.7	< 0.001
Female	23.8 (1663/6973)	22.8 (2051/8993)	0.12
Clinical presentation			
Chronic coronary syndromes	51.6 (3601/6973)	54.3 (4880/8993)	0.001
Acute coronary syndromes			
Unstable angina	12.2 (852/6973)	13.0 (1169/8993)	
NSTEMI	21.1 (1473/6973)	21.1 (1900/8993)	
STEMI	15.0 (1047/6973)	11.6 (1044/8993)	
Comorbidities			
Diabetes mellitus	18.6 (1293/6968)	30.5 (2745/8987)	< 0.001
Insulin treated	5.3 (367/6956)	9.6 (856/8963)	< 0.001
Hypertension	67.3 (4677/6947)	78.5 (7038/8965)	< 0.001
Hypercholesterolemia	65.9 (4446/6751)	72.6 (6322/8712)	< 0.001
Current smoker	28.3 (1973/6973)	24.4 (2195/8993)	< 0.001
PVD	7.1 (488/6904)	5.8 (517/8916)	0.001
COPD	4.8 (336/6938)	5.4 (485/8956)	0.11
Renal impairment^*^	12.3 (856/6936)	14.7 (1315/8945)	< 0.001
Medical history			
Previous bleeding	0.7 (46/6966)	0.6 (52/8979)	0.52
Previous stroke	2.4 (167/6960)	2.8 (254/8983)	0.09
Previous MI	22.0 (1530/6952)	24.3 (2180/8968)	0.001
Previous PCI	30.9 (2152/6968)	34.2 (3069/8984)	< 0.001
Previous CABG	5.8 (406/6967)	6.0 (537/8986)	0.69
Procedure			
Radial access	73.4 (5089/6931)	74.5 (6670/8950)	0.12
Number of lesions treated			0.36
One lesion	68.0 (4698/6913)	68.2 (6094/8930)	
Two lesions	22.8 (1575/6913)	23.1 (2066/8930)	
Three or more	9.3 (640/6913)	8.6 (770/8930)	
Average number	1.4 ± 0.8	1.4 ± 0.7	0.18
Left main PCI	2.9 (198/6913)	2.6 (231/8930)	0.29
RCA PCI	37.7 (2607/6913)	37.5 (3347/8930)	0.77
LAD PCI	51.7 (3575/6913)	50.1 (4476/8930)	0.047
LCX PCI	30.7 (2125/6913)	32.3 (2884/8930)	0.037
Bypass graft PCI	1.4 (94/6913)	1.4 (124/8930)	0.88
Multivessel PCI	22.9 (1583/6913)	22.3 (1991/8930)	0.37

Data are presented as mean ± standard deviation or percentage (number)

^*^Based on creatinine-estimated GFR (eGFR) clearance of < 60 ml/min/1.73 m^2^, using the Modification of Diet in Renal Disease (MDRD) formula.

*BMI* body mass index, *PVD* peripheral vascular disease, *COPD* chronic obstructive pulmonary disease, *MI* myocardial infarction, *STEMI* ST-elevation myocardial infarction, *NSTEMI* Non-STEMI, *PCI* percutaneous coronary intervention, *CABG* coronary artery bypass graft; RCA: right coronary artery, *LAD* left anterior descending artery, *LCX* left circumflex artery

Baseline patient characteristics were comparable and well balanced between the experimental and reference arms in each BMI group as shown in online Table 1.

### Comparison of 2-year clinical outcomes between BMI groups

In a univariate analysis, patients with a BMI < 27 kg/m^2^ had at 2 years follow-up a higher rate of the primary endpoint (4.4% vs. 3.8%, unadjusted HR 1.17, 95% CI 1.00–1.37, *p* = 0.044) and secondary safety endpoint (2.4% vs. 1.9%, unadjusted HR 1.27, 95% CI 1.02–1.57, *p* = 0.033) compared with those with BMI ≥ 27 kg/m^2^ (Table 2).

**Table 2:** Clinical outcomes with unadjusted and adjusted hazard ratios between patients with BMI < 27 kg/m^2^ and ≥ 27 kg/m^2^

Outcomes at 2 years	BMI < 27 kg/m^2^	BMI ≥ 27 kg/m^2^	Unadjusted HR; BMI < 27/BMI ≥ 27		Adjusted HR; BMI < 27/BMI ≥ 27	
	No. (%)	No. (%)	(95%CI)	*p* value	(95% CI)	*P * value
All-cause death or new Q-wave MI	310 (4.4)	343 (3.8)	1.17 (1.00–1.37)	0.044	1.14 (0.97–1.34)	0.12
All-cause death	236 (3.4)	241 (2.7)	1.27 (1.06–1.52)	0.009	1.24 (1.02–1.49)	0.029
New Q wave MI	80 (1.1)	106 (1.2)	0.98 (0.73–1.31)	0.88	0.94 (0.69–1.28)	0.70
All-cause death, stroke, or new Q-wave MI	366 (5.2)	412 (4.6)	1.15 (1.00–1.32)	0.051	1.13 (0.98–1.32)	0.10
BARC 3 or 5 bleeding	164 (2.4)	168 (1.9)	1.27 (1.02–1.57)	0.030	1.10 (0.88–1.37)	0.42
BARC 5 bleeding	20 (0.3)	26 (0.3)	1.00 (0.56–1.79)	0.99	0.74 (0.40–1.37)	0.34
BARC 3 bleeding	153 (2.2)	156 (1.7)	1.27 (1.02–1.59)	0.033	1.12 (0.89–1.41)	0.34
BARC 2 bleeding	338 (4.8)	447 (5.0)	0.98 (0.85–1.13)	0.79	0.92 (0.79–1.06)	0.24
Definite stent thrombosis	52 (0.7)	76 (0.8)	0.89 (0.62–1.26)	0.50	0.91 (0.63–1.31)	0.61

Data are presented as number (%). Unadjusted and adjusted hazard ratios (95% confidential interval) are derived from univariate and multivariate Cox regression model, respectively. Adjusted covariates for bleeding events (BARC type 3 or 5 bleeding, those components, and BARC type 2 bleeding) are age (years), sex, clinical presentation (CCS or ACS), diabetes mellitus, previous bleeding, renal impairment, anemia according to WHO classification, and radial access in the index procedure. Adjusted covariates for other outcomes are age (years), sex, clinical presentation (CCS or ACS), diabetes mellitus, hypertension, hypercholesteremia, PVD, COPD, renal impairment, previous MI, previous PCI, and previous CABG

*BARC* Bleeding Academic Research Consortium; *WHO* World Health Organization; Other abbreviations as in Table 1

For the multivariable analysis, the VIF values of covariables were all < 2.0, indicating no evidence for strong multicollinearity. After adjustment for potential confounding factors, the risk of all-cause death at 2 years remained higher in patients with BMI < 27 kg/m^2^ than in patients with BMI ≥ 27 kg/m^2^ (3.4% vs. 2.7%, unadjusted HR 1.27, 95% CI 1.06–1.52, *p* = 0.009, adjusted HR 1.24, 95% CI 1.02–1.49, *p* = 0.029), but other clinical outcomes including the primary (adjusted HR 1.14, 95% CI 0.97–1.34, *p* = 0.12) and secondary endpoint (adjusted HR 1.10, 95% CI 0.88–1.37, *p* = 0.42) were no longer significantly different between the two BMI groups (Table 2).

The comparison of clinical outcomes according to WHO classification is shown in online Table 2. After adjusting confounding factors, the risk of all-cause mortality at 2 years was significantly lower in overweight patients (HR 0.75, 95% CI 0.60–0.93, *p* = 0.010) or obese patients (HR 0.74, 95% CI 0.57–0.95, *p* = 0.020) than normal weight patients. The correlation between the risks for the primary or secondary endpoint and BMI as a continuous variable showed reverse J-shape curves, as shown in Fig. 2 and online Table 3. The ROC analysis demonstrated that 25.4 kg/m^2^ was the optimal cutoff value of BMI for predicting the primary endpoint.

### Impact of BMI on antiplatelet strategy

**Fig. 3:**
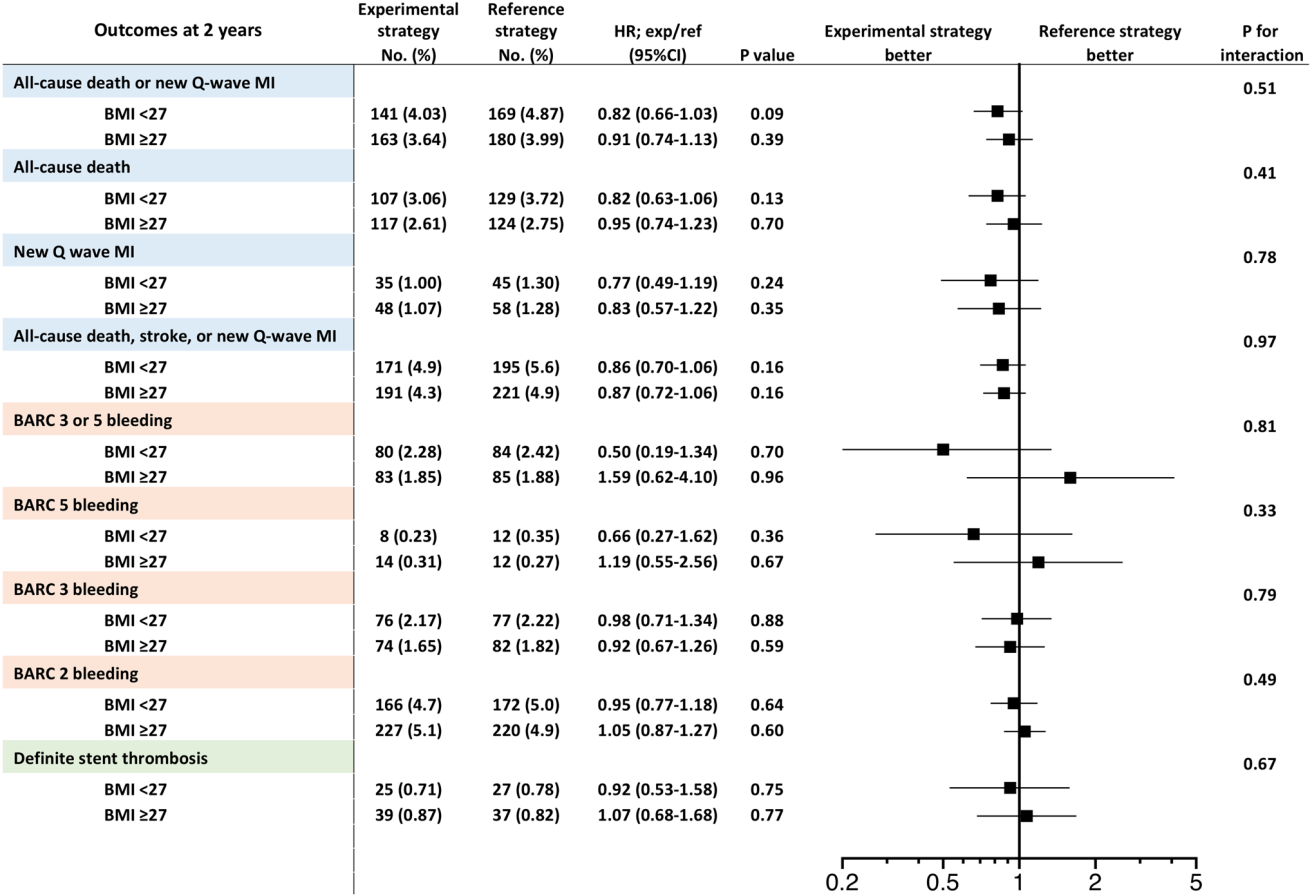
Clinical outcomes at 2-year and forest plots in comparison of patients stratified according to BMI with threshold of 27 kg/m^2^. The squares indicate estimated hazard ratio, and the horizontal lines indicate 95% CI. There was no statistically significant difference in any clinical outcomes between experimental strategy and reference strategy in each BMI group (BMI < 27 kg/m^2^ or ≥ 27 kg/m^2^). *p*_interaction_ values were derived from Cox regression model. Abbreviations as in Fig. 2

The comparison of 2-year outcomes between the experimental and reference arms are shown in Fig. 3. At the 2-year follow-up, there was no statistically significant treatment effect on the primary endpoint of all-cause mortality or new Q-wave MI between the experimental and reference arm in patients with a BMI < 27 kg/m^2^ (4.9% vs. 4.0%, HR 0.82, 95% CI 0.66–1.03, *p* = 0.09), or BMI ≥ 27 kg/m^2^ (4.0% vs. 3.6%, HR0.91, 95% CI0.74–1.13, *p* = 0.39, *p*_interaction_ = 0.51). Similarly, there was no significant effect between the antiplatelet strategies on the secondary endpoint of BARC type 3 or 5 bleeding for either BMI group (BMI < 27 kg/m^2^, 2.3% vs. 2.4%, HR 0.94, 95% CI 0.69–1.28, *p* = 0.70, BMI ≥ 27 kg/m^2^, 1.9% vs. 1.9%, HR 0.99, 95% CI 0.73–1.34, *p* = 0.96, *p*_interaction_ = 0.81). There was no beneficial treatment effect related to the experimental strategy with ticagrelor monotherapy with regard to other clinical outcomes at 2 years in each BMI group (Fig. 3).

### Comparison of clinical outcomes between the two antiplatelet strategies in each BMI group stratified according to their clinical presentation

Clinical outcomes stratified according to clinical presentation (ACS or CCS) and BMI (< 27 or ≥ 27 kg/m^2^) are shown in Table 3.

### Impact of BMI on antiplatelet strategy in the setting of ACS

In the patients with ACS and a BMI < 27 kg/m^2^, the experimental antiplatelet strategy resulted in a significantly lower rate of the primary endpoint of all-cause mortality or new Q-wave MI compared to the reference arm (5.8% vs. 4.1%, HR0.69, 95% CI0.51–0.94, *p* = 0.019) with a significant treatment effect (*p*_interaction_ = 0.047, Table 3), which was not seen in those with a BMI ≥ 27 kg/m^2^ (3.8% vs. 3.5%, HR 1.09, 95% CI 0.79–1.50, *p* = 0.60). The secondary safety bleeding endpoint (BARC type 3 or 5 bleeding) was numerically lower in patients with ACS and a BMI < 27 kg/m^2^ receiving the experimental regime; however, there was no significant treatment effect (2.1% vs. 3.0%, HR 0.69, 95% CI 0.45–1.06, *p* = 0.09, *p*_interaction_ = 0.75) (Table 3). In patients with ACS and a BMI ≥ 27 kg/m^2^, there was no significant difference in the incidence of the secondary safety bleeding endpoint between the treatment arms (1.8% vs. 2.4%, HR 0.76, 95% CI 0.50–1.17, *p* = 0.21, *p*_interaction_ = 0.75), whereas BARC 3 bleeding was significantly lower in the experimental arm than in the reference arm (1.5% vs. 2.4%, HR 0.62, 95% CI 0.39–0.97, *p* = 0.038), yet without *p* value for interaction (*p*_interaction_ = 0.59).

**Table 3:** Clinical outcomes at 2 years in comparison between reference and experimental antiplatelet strategy stratified according to BMI and clinical presentation

Outcomes at 2 years	Acute coronary syndromes (*N* = 7485, 46.9%)					Chronic coronary syndromes (N = 8481, 53.1%)				
	Reference strategy no. (%)	Experimental strategy no. (%)	HR; exp/ref. (95% CI)	*p* value	*p*_interaction_	Reference strategy No. (%)	Experimental strategy No. (%)	HR; exp/ref (95%CI)	*P *value	*P *interaction
All-cause death or new Q-wave MI										
BMI < 27 kg/m^2^	97 (5.8)	69 (4.1)	0.69 (0.51–0.94)	0.019	0.047	72 (4.0)	72 (4.0)	0.99 (0.72–1.38)	0.98	0.31
BMI ≥ 27 kg/m^2^	72 (3.5)	78 (3.8)	1.09 (0.79–1.50)	0.60		108 (4.4)	85 (3.5)	0.79 (0.60–1.06)	0.11	
All-cause death										
BMI < 27 kg/m^2^	79 (4.7)	57 (3.4)	0.71 (0.50–0.99)	0.045	0.07	50 (2.8)	50 (2.8)	1.00 (0.67–1.47)	0.98	0.49
BMI ≥ 27 kg/m^2^	53 (2.6)	59 (2.9)	1.12 (0.77–1.62)	0.55		71 (2.9)	58 (2.4)	0.83 (0.58–1.17)	0.28	
New Q wave MI										
BMI < 27 kg/m^2^	22 (1.3)	13 (0.8)	0.58 (0.29–1.15)	0.12	0.20	23 (1.3)	22 (1.2)	0.95 (0.53–1.71)	0.87	0.48
BMI ≥ 27 kg/m^2^	19 (0.9)	20 (1.0)	1.06 (0.56–1.98)	0.86		39 (1.6)	28 (1.2)	0.73 (0.45–1.18)	0.19	
All-cause death, stroke, or new Q-wave MI										
BMI < 27 kg/m^2^	108 (6.4)	84 (4.9)	0.76 (0.57–1.00)	0.058	0.26	87 (4.8)	87 (4.8)	1.00 (0.74–1.34)	0.97	0.29
BMI ≥ 27 kg/m^2^	94 (4.6)	90 (4.4)	0.96 (0.72–1.28)	0.78		127 (5.2)	101 (4.2)	0.80 (0.62–1.04)	0.10	
BARC 3 or 5 bleeding										
BMI < 27 kg/m^2^	51 (3.0)	36 (2.1)	0.69 (0.45–1.06)	0.09	0.75	33 (1.8)	44 (2.4)	1.33 (0.85–2.09)	0.21	0.95
BMI ≥ 27 kg/m^2^	49 (2.4)	37 (1.8)	0.76 (0.50–1.17)	0.21		36 (1.5)	46 (1.9)	1.31 (0.84–2.02)	0.23	
BARC 5 bleeding										
BMI < 27 kg/m^2^	6 (0.4)	3 (0.2)	0.49 (0.12–1.97)	0.32	0.17	6 (0.3)	5 (0.3)	0.83 (0.25–2.72)	0.76	0.75
BMI ≥ 27 kg/m^2^	7 (0.3)	11 (0.5)	1.59 (0.62–4.10)	0.34		5 (0.2)	3 (0.1)	0.61 (0.15–2.56)	0.50	
BARC 3 bleeding										
BMI < 27 kg/m^2^	48 (2.9)	36 (2.1)	0.73 (0.48–1.13)	0.16	0.59	29 (1.6)	40 (2.2)	1.38 (0.85–2.22)	0.19	0.97
BMI ≥ 27 kg/m^2^	49 (2.4)	30 (1.5)	0.62 (0.39–0.97)	0.038		33 (1.3)	44 (1.8)	1.36 (0.87–2.14)	0.18	
BARC 2 bleeding										
BMI < 27 kg/m^2^	103 (6.1)	82 (4.8)	0.77 (0.58–1.03)	0.08	0.18	69 (3.8)	84 (4.7)	1.21 (0.88–1.67)	0.23	0.58
BMI ≥ 27 kg/m^2^	105 (5.1)	105 (5.1)	0.91 (0.77–1.33)	0.91		115 (4.7)	122 (5.0)	1.08 (0.84–1.40)	0.54	
Definite stent thrombosis										
BMI < 27 kg/m^2^	16 (1.0)	8 (0.5)	0.49 (0.21–1.15)	0.10	0.10	11 (0.6)	17 (0.9)	1.54 (0.72–3.30)	0.26	0.36
BMI ≥ 27 kg/m^2^	21 (1.0)	24 (1.2)	1.16 (0.64–2.07)	0.63		16 (0.7)	15 (0.6)	0.95 (0.47–1.93)	0.90	

Data are presented as number (%). *p*_interaction_ values were derived from Cox regression model

*BARC* Bleeding Academic Research Consortium, *exp* experimental strategy, *ref* reference strategy, *NA* not applicable, other abbreviations as in Table 1

**Fig. 4:**
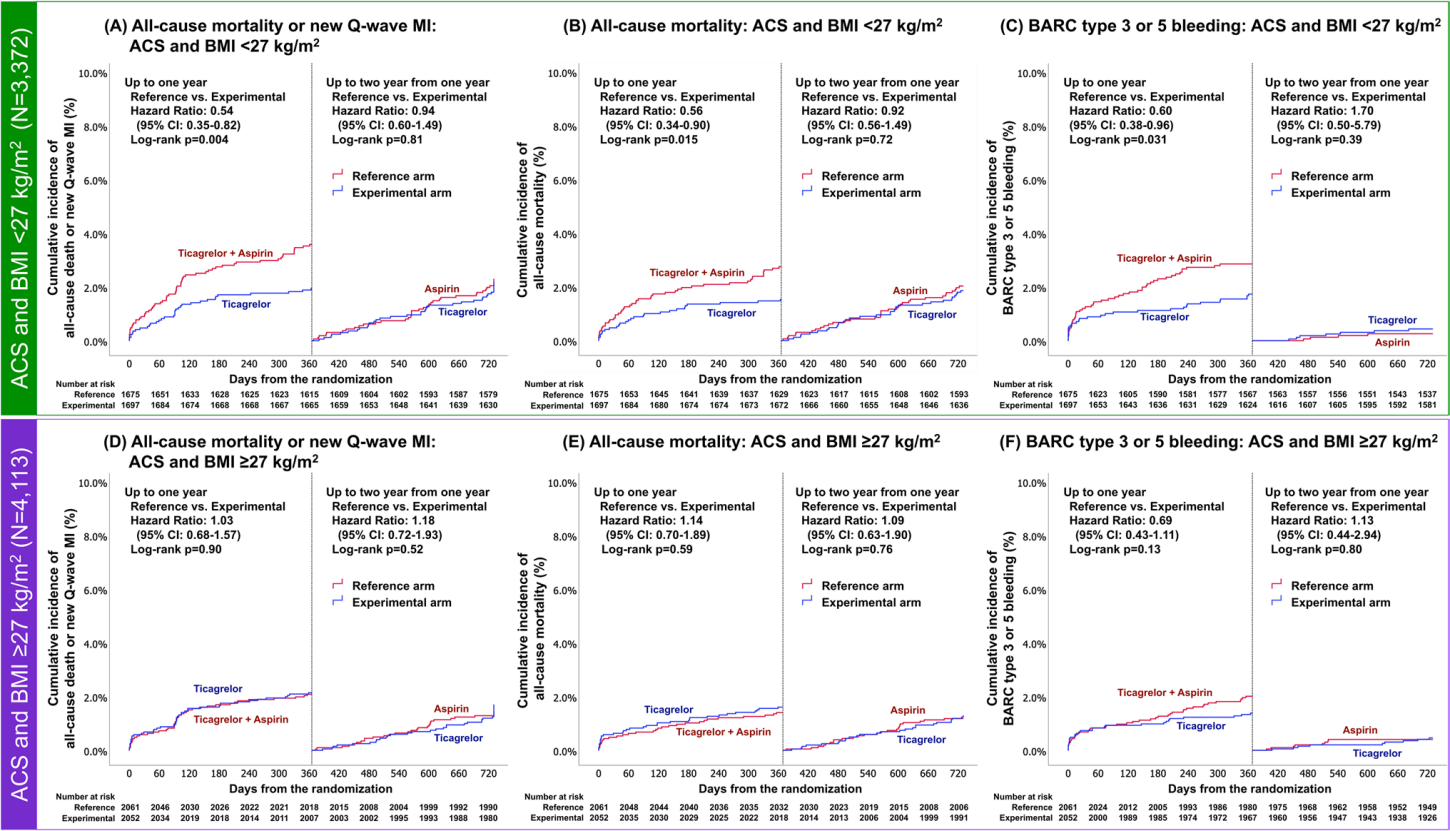
The 1-year landmark analysis and Kaplan–Meier curves in patients with ACS and either BMI < 27 kg/m^2^ or BMI ≥ 27 kg/m^2^. The 1-year landmark analyses of primary endpoint (all-cause mortality or new Q-wave MI), all-cause mortality, and secondary safety endpoint (BARC type 3, or 5 bleeding) have demonstrated that the reduced risks of adverse events in experimental arm compared to reference arm were largely obtained at 1 year in patients with ACS and BMI < 27 kg/m^2^. However, in patients with ACS and BMI ≥ 27 kg/m^2^, no treatment benefits were seen in terms of primary endpoint, all-cause mortality, and BARC type 3 or 5 bleeding, either in the first-year and up to 2 years from 1 year. Abbreviations as in Fig. 2

In patients with BMI < 27 kg/m^2^ and ACS, the observed lower rates of events with the experimental treatment were mainly driven by the lower incidence of all-cause mortality, BARC 3 or 5 bleeding, or BARC 2 bleeding during the first year after index PCI; a landmark analysis after 1 year did not show any treatment effect in the second year (Fig. 4 and Suppl. Fig. 1).

### Impact of BMI on antiplatelet strategy in the setting of CCS

In the setting of CCS, there was no difference between the reference and the experimental arm regardless of BMI group in terms of the primary endpoint (BMI < 27 kg/m^2^; 4.0% vs. 4.0%, HR 0.99, 95% CI 0.72–1.38, *p* = 0.98; BMI ≥ 27 kg/m^2^; 3.5% vs. 4.4%, HR 0.79, 95% CI 0.60–1.06, *p* = 0.11, *p*_interaction_ = 0.31) nor the secondary endpoint (BMI < 27 kg/m^2^; 2.4% vs. 1.8%, HR 1.33, 95% CI 0.85–2.09, *p* = 0.21; BMI ≥ 27 kg/m^2^; 1.9% vs. 1.5%, HR 1.31, 95% CI 0.84–2.02, *p* = 0.23, *p*_interaction_ = 0.95) (Table 3).

## Discussion

In the context of a neutral trial, all presented findings should be viewed strictly as hypothesis generating. Nevertheless, for the first time to our knowledge, we have observed a differential effect of ticagrelor monotherapy, when compared with ticagrelor and aspirin, in relation to baseline BMI in patients with ACS—a subgroup who between 31 and 365 days after randomization were assigned to receive either ticagrelor alone, or in combination with aspirin by the GLOBAL LEADERS trial protocol [14].

In the present study, the potential beneficial effect of the experimental strategy was only observed in patients with ACS who had a BMI < 27 kg/m^2^, and was not seen in those with higher BMIs. Platelet hyper-reactivity and activation plays a central role in the progression of atherothrombosis and is the result of interactions of many adaptive responses to obesity: insulin resistance, inflammation, oxidative stress, and endothelial dysfunction [2, 26].

Although a plausible pharmacodynamic explanation still needs to be determined, it can be explained by some hypothesis. Patients with high BMI and ACS are more likely to have a prothrombotic state, partly linked to dysglycemia and proinflammatory effects of metabolic syndrome. In the PLATO study, the beneficial effect of potent antiplatelet regimen with ticagrelor was mainly observed when the patient’s body weight was higher than the median value for their sex (*p*_interaction_ = 0.04) [27] In addition, the substudy of the PLATO trial showed that impaired fibrinolysis was an independent predictor of cardiovascular death and was more common in patients with diabetes mellitus and/or higher BMI [28]. In those situations, strong agonist stimulation such as via platelet thrombin receptors as well as via collagen-mediated thromboxane A2 release could overwhelm the effects of potent platelet P2Y12 inhibition.

Furthermore, among obese patients, cyclo-oxygenase (COX) inhibition, which is achieved exclusively by aspirin, may play a more vital role than in non-obese patients. It has been demonstrated that excess adipose tissue is associated with an increased platelet turnover, leading to unacetylated COX-1 and COX-2 in newly formed platelets with subsequent excessive thromboxane formation [29, 30]. This is further exacerbated by extra-platelet sources of thromboxane in obese patients driven by inflammatory triggers and enhanced lipid peroxidation, resulting in activation of platelets by a mechanism bypassing COX-1 acetylation or through limiting COX-isozyme acetylation by aspirin [29, 30]. Consequently, ticagrelor monotherapy may provide insufficient antithrombotic effect compared to ticagrelor plus aspirin in obese patients with prothrombotic states [31, 32]. In other words, it is possible that the balance of inhibition of platelet thromboxane A_2_ (TXA_2_) release vs inhibition of prostacyclin formation with standard DAPT regimens is more favorable in obese patients than in non-obese patients [33]. More than a decade ago, and before the availability of prasugrel and ticagrelor, high BMI was associated with stent thrombosis in the all-comers LEADERS trial, leading to calls for the dose of clopidogrel to be weight adjusted [34].

On the other hand, in patients with ACS and a BMI < 27 kg/m^2^, the potentially favorable results of ticagrelor monotherapy compared to DAPT during the first year require some cautious interpretation. Previously, Leadbeater, et al. and Kirkby, et al. demonstrated that sufficient inhibition of the TXA_2_ pathway can be achieved with the sole use of a strong P2Y_12_ inhibitor such as prasugrel or ticagrelor without aspirin [35]; however, these findings were not seen consistently [36, 37], although, this may have been due to the heterogeneity of the studied populations. Whereas the possibility of a play of chance remains, our results might suggest that in non-obese patients with higher responsiveness to P2Y_12_ inhibitors [38, 39], sufficient inhibition of TXA_2_ pathway could be achieved by ticagrelor monotherapy, and adding aspirin could be associated with higher risks of ischemic and bleeding events than in obese patients [40]. In summary, the BMI-adjusted antiplatelet strategy with or without aspirin may be effective in ACS patients undergoing PCI, and the aspirin-free strategy with a potent P2Y12 inhibitor could be beneficial for those with a relatively low BMI.

In patients with CCS, the experimental strategy resulted in no significant difference in any clinical outcomes, but did lead to numerically higher rates of major bleeding in patients irrespective of their BMI group. Although Orme et al. reported that lower platelet activity achieved with ticagrelor, compared with clopidogrel, also occurred in patients with CCS [41], our results might suggest that the anti-ischemic effect of potent P2Y_12_ inhibitors may not be required in low ischemic-risk settings such as patients with CCS.

Finally, in our cohort, and consistent with previous studies, we observed the “obesity paradox” with the reverse J-shape association between adverse events and BMI as a continuous variable [6, 42, 43]. In addition, normal weight patients had a higher risk of all-cause mortality compared with overweight or obese patients according to the WHO classification (Table 2). Given the fact that most patients with a BMI < 27 kg/m^2^ in this study could be categorized as “normal weight” in the WHO classification (Fig. 2), our results may encourage the efficacy of the novel P2Y12 inhibitor monotherapy for those high-risk “normal weight” patients.

## Limitations

The present study needs to be interpreted in light of the following limitations. First, the present study consists of two prespecified subgroup analyses of a randomized controlled study with multiple testing (BMI and clinical presentation). Because in the GLOBAL LEADERS trial two different P2Y_12_ inhibitors are used in the reference group depending on the clinical presentation of ACS (ticagrelor) or CCS (clopidogrel), multiple analyses according to the clinical presentation have to be performed to evaluate specifically the treatment effect strictly. However, the results could be a play of chance and they should be considered as hypothesis generating. Second, BMI data were only available at the time of randomization. BMI can change depending on weight gain or loss during follow-up [44]. Third, in past trials reporting the “obesity paradox”, the current threshold of BMI (27 kg/m^2^) prespecified in the design paper and based on a recent publication [16] was not widely used and was higher than the optimal cutoff value of 25.4 kg/m^2^ for stratifying with the risk of the primary endpoint in this study. In addition, the WHO classification is somewhat different. Indeed, the WHO classification classified patients into four or six categories, resulting in lower and uneven statistical power among these groups. Our threshold was close to the median value of 27.68 kg/m^2^ in the current study, which allows uniform statistical power in each group. Fourth, in this trial all endpoints were site reported without a clinical adjudication committee for serious adverse events due to limited financial resources. However, the GLASSY study [45], which is a prespecified ancillary study of the GLOBAL LEADERS trial with event adjudication by an independent clinical event committee, confirmed the consistent results with those of site reported.

## Conclusion

There was no overall treatment effect of experimental ticagrelor monotherapy versus standard DAPT strategy between the groups with high or low baseline BMI. However, a beneficial treatment effect on ischemic events (primary endpoints of all-cause mortality or new Q-wave MI) without trade-off in bleeding (BARC type 3 or 5 bleeding) of the experimental treatment with ticagrelor monotherapy was observed in patients presenting with ACS with BMI < 27 kg/m^2^, which was not seen in patients with BMI ≥ 27 kg/m^2^. Our results suggest the potential benefit of a novel antiplatelet monotherapy regimen in targeting non-obese ACS patients.

## Electronic supplementary material

Below is the link to the electronic supplementary material.

Supplementary file1 (DOCX 7246 kb)

## Abbreviations

ACS: Acute coronary syndromes
BARC: Bleeding Academic Research Consortium
BMI: Body mass index
CCS: Chronic coronary syndromes
DAPT: Dual antiplatelet therapy
DES: Drug-eluting stent
MI: Myocardial infarction
PCI: Percutaneous coronary intervention
