# Supplementary material for: The association of body mass index with long-term clinical outcomes after ticagrelor monotherapy following abbreviated dual antiplatelet therapy in patients undergoing percutaneous coronary intervention: a prespecified sub-analysis of the GLOBAL LEADERS Trial
Source: Clin Res Cardiol. 2020 Jan 31;109(9):1125–39. doi: 10.1007/s00392-020-01604-1 (PMC7449952; doi:10.1007/s00392-020-01604-1)
Supplement: Supplementary file 1 — Supplementary file1 (DOCX 7246 kb) [file 392_2020_1604_MOESM1_ESM.docx]

**Supplementary Appendix**

**The Association of Body Mass Index with Long-term Clinical Outcomes after Ticagrelor Monotherapy Following Abbreviated Dual Antiplatelet Therapy in Patients undergoing Percutaneous Coronary Intervention: Insights from the GLOBAL LEADERS trial**

**Suppl. Fig. 1 The one-year landmark analysis and Kaplan-Meier curves in patients with ACS and either BMI <27 kg/m^2^ or BMI ≥27 kg/m^2^**

The one-year landmark analyses of BARC type 2, 3, or 5 bleeding and those components demonstrated that the reduced risks of bleeding events in experimental arm compared to reference arm were largely obtained at one year in patients with ACS and BMI <27kg/m^2^ (A, B, C, and D). Whereas, in patients with ACS and BMI >27 kg/m^2^, only BARC type 3 bleeding up to 1-year showed the reduction of risk in the experimental strategy, but no other treatment benefits were seen in terms of bleeding events, either in the first-year and up to 2-year from 1-year (E, F, G, and H). Abbreviations as in Figure 2.

**Online Table 1. Clinical and angiographic characteristics of patients between reference arm and experimental arm stratified according BMI**

|  | BMI <27 | | | BMI ≥27 | | | | | |
| --- | --- | --- | --- | --- | --- | --- | --- | --- | --- |
|  | N=6973/15966 (43.7%) | | | N=8993/15996 (56.3%) | | | | | |
|  | Reference strategy | Experimental strategy | p | Reference strategy | | Experimental strategy | | p | |
|  | N=3471 (49.8%) | N=3502 (50.2%) | value | N=4516 (50.2%) | | N=4477 (49.8%) | | value | |
| Age (years) | 65.7±10.5 | 65.5±10.5 | 0.35 | 63.7±10.0 | 63.8±10.1 | | 0.78 | |  |
| BMI (kg/m^2^) | 24.3±2.0 | 24.3±2.0 | 0.38 | 31.2±3.8 | 31.2±3.7 | | 0.97 | |  |
| Female | 23.6 (818/ 3471) | 24.1 (845/ 3502) | 0.58 | 22.8 (1031/ 4516) | 22.8 (1020/ 4477) | | 0.96 | |  |
| Clinical presentation |  |  |  |  |  | |  | |  |
| Chronic Coronary Syndromes | 51.7 (1796/ 3471) | 51.5 (1805/ 3502) | 0.87 | 54.4 (2455/ 4516) | 54.2 (2425/ 4477) | | 0.85 | |  |
| Acute Coronary Syndromes |  |  |  |  |  | |  | |  |
| Unstable angina | 12.4 (429/ 3471) | 12.1 (423/ 3502) |  | 13.0 (589/ 4516) | 13.0 (580/ 4477) | |  | |  |
| NSTEMI | 20.9 (727/ 3471) | 21.3 (746/ 3502) |  | 21.3 (962/ 4516) | 21.0 (938/ 4477) | |  | |  |
| STEMI | 15.0 (519/ 3471) | 15.1 (528/ 3502) |  | 11.3 (510/ 4516) | 11.9 (534/ 4477) | |  | |  |
| Comorbidities |  |  |  |  |  | |  | |  |
| Diabetes mellitus | 18.3 (635/ 3469) | 18.8 (658/ 3499) | 0.59 | 30.0 (1354/ 4513) | 31.1 (1391/ 4474) | | 0.26 | |  |
| Insulin-treated | 5.1 (176/ 3465) | 5.5 (191/ 3491) | 0.46 | 9.8 (441/ 4500) | 9.3 (415/ 4463) | | 0.42 | |  |
| Hypertension | 67.2 (2324/ 3458) | 67.4 (2353/ 3489) | 0.84 | 78.0 (3509/ 4501) | 79.1 (3529/ 4464) | | 0.21 | |  |
| Hypercholesterolemia | 65.8 (2210/ 3361) | 66.0 (2236/ 3390) | 0.86 | 73.3 (3213/ 4385) | 71.9 (3109/ 4327) | | 0.14 | |  |
| Current smoker | 28.4 (986/ 3471) | 28.2 (987/ 3502) | 0.84 | 24.7 (1117/ 4516) | 24.1 (1078/ 4477) | | 0.47 | |  |
| PVD | 7.6 (263/ 3439) | 6.5 (225/ 3465) | 0.06 | 5.9 (266/ 4478) | 5.7 (251/ 4438) | | 0.57 | |  |
| COPD | 5.0 (171/ 3454) | 4.7 (165/ 3484) | 0.68 | 5.5 (246/ 4494) | 5.4 (239/ 4462) | | 0.81 | |  |
| Renal impairment^*^ | 12.1 (419/ 3453) | 12.5 (437/ 3483) | 0.60 | 14.5 (653/ 4495) | 14.9 (662/ 4450) | | 0.64 | |  |
| Medical history |  |  |  |  |  | |  | |  |
| Previous bleeding | 0.7 (24/ 3467) | 0.6 (22/ 3499) | 0.74 | 0.6 (28/ 4511) | 0.5 (24/ 4468) | | 0.60 | |  |
| Previous stroke | 2.3 (80/ 3465) | 2.5 (87/ 3495) | 0.62 | 2.9 (131/ 4512) | 2.8 (123/ 4471) | | 0.66 | |  |
| Previous MI | 22.7 (786/ 3461) | 21.3 (744/ 3491) | 0.16 | 24.3 (1093/ 4504) | 24.4 (1087/ 4464) | | 0.93 | |  |
| Previous PCI | 31.2 (1082/ 3467) | 30.6 (1070/ 3501) | 0.56 | 33.9 (1530/ 4512) | 34.4 (1539/ 4472) | | 0.61 | |  |
| Previous CABG | 6.1 (212/ 3466) | 5.5 (194/ 3501) | 0.31 | 6.3 (283/ 4514) | 5.7 (254/ 4472) | | 0.24 | |  |
| Procedure |  |  |  |  |  | |  | |  |
| Radial access | 73.2 (2523/ 3446) | 73.6 (2566/ 3485) | 0.70 | 74.9 (3365/ 4493) | 74.2 (3305/ 4457) | | 0.42 | |  |
| Number of lesions treated |  |  | 0.13 |  |  | | 0.94 | |  |
| One lesion | 68.4 (2351/ 3436) | 67.5 (2347/ 3477) |  | 68.4 (3068/ 4485) | 68.1 (3026/ 4445) | |  | |  |
| Two lesions | 21.9 (752/ 3436) | 23.7 (823/ 3477) |  | 23.1 (1034/ 4485) | 23.2 (1032/ 4445) | |  | |  |
| Three or more | 9.7 (333/ 3436) | 8.8 (307/ 3477) |  | 8.5 (383/ 4485) | 8.7 (387/ 4445) | |  | |  |
| Average number | 1.45±0.77 | 1.44±0.75 | 0.86 | 1.43±0.73 | 1.43±0.73 | | 0.79 | |  |
| Left main PCI | 2.9 (98/ 3436) | 2.9 (100/ 3477) | 0.95 | 2.5 (111/ 4485) | 2.7 (120/ 4445) | | 0.50 | |  |
| RCA PCI | 30.6 (1052/ 3436) | 30.9 (1073/ 3477) | 0.83 | 32.3 (1447/ 4485) | 32.3 (1437/ 4445) | | 0.95 | |  |
| LAD PCI | 52.4 (1802/ 3436) | 51.0 (1773/ 3477) | 0.23 | 50.9 (2281/ 4485) | 49.4 (2195/ 4445) | | 0.16 | |  |
| LCX PCI | 37.1 (1274/ 3436) | 38.3 (1333/ 3477) | 0.28 | 36.7 (1647/ 4485) | 38.2 (1700/ 4445) | | 0.14 | |  |
| Bypass graft PCI | 1.3 (44/ 3436) | 1.4 (50/ 3477) | 0.57 | 1.4 (62/ 4485) | 1.4 (62/ 4445) | | 0.96 | |  |
| Multivessel PCI | 22.7 (779/ 3436) | 23.1 (804/ 3477) | 0.65 | 22.2 (994/ 4485) | 22.4 (997/ 4445) | | 0.76 | |  |
|  |  |  |  |  |  | |  | |  |

Data are presented as mean ± standard deviation or percentage (number).

* Based on creatinine-estimated GFR (eGFR) clearance of <60 ml/min/1.73 m^2^, using the Modification of Diet in Renal Disease (MDRD) formula.

BMI: body mass index; PVD: peripheral vascular disease; COPD: chronic obstructive pulmonary disease; MI: myocardial infarction; STEMI: ST-elevation myocardial infarction; NSTEMI: Non-STEMI; PCI: percutaneous coronary intervention; CABG: coronary artery bypass graft; RCA: right coronary artery; LAD: left anterior descending artery; LCX: left circumflex artery.

**Online Table 2. Clinical outcomes and hazard ratios among four groups according to WHO classification**

| Outcomes at 2 years | Underweight N=79 | Normal weight N=3,902 | Overweight N=7,223 | Obesity N=4,762 | Adjusted HR; Overweight/Normal weight |  | Adjusted HR; Obesity/Normal weight |  |
| --- | --- | --- | --- | --- | --- | --- | --- | --- |
|  | No. (%) | No. (%) | No. (%) | No. (%) | (95% CI) | P value | (95% CI) | P value |
| All-cause death or new Q-wave MI | 7 (8.9) | 186 (4.8) | 281 (3.9) | 179 (3.8) | 0.84 (0.69-1.01) | 0.07 | 0.83 (0.67-1.04) | 0.10 |
| All-cause death | 7 (8.9) | 148 (3.8) | 197 (2.7) | 125 (2.6) | 0.75 (0.60-0.93) | 0.010 | 0.74 (0.57-0.95) | 0.020 |
| New Q wave MI | 0 (0) | 43 (1.1) | 87 (1.2) | 56 (1.2) | 1.08 (0.74-1.58) | 0.69 | 1.08 (0.71-1.64) | 0.73 |
| All-cause death, stroke, or new Q-wave MI | 9 (11.4) | 218 (5.6) | 333 (4.6) | 218 (4.6) | 0.84 (0.70-1.00) | 0.054 | 0.84 (0.69-1.02) | 0.08 |
| BARC 3 or 5 bleeding | 7 (8.9) | 92 (2.4) | 148 (2) | 85 (1.8) | 0.96 (0.74-1.26) | 0.77 | 0.92 (0.67-1.25) | 0.58 |
| BARC 5 bleeding | 0 (0) | 15 (0.4) | 20 (0.3) | 11 (0.2) | 0.95 (0.47-1.92) | 0.89 | 0.89 (0.39-2.03) | 0.79 |
| BARC 3 bleeding | 7 (8.9) | 84 (2.2) | 139 (1.9) | 79 (1.7) | 0.98 (0.74-1.29) | 0.86 | 0.91 (0.66-1.25) | 0.56 |
| BARC 2 bleeding | 3 (3.8) | 190 (4.9) | 359 (5) | 233 (4.9) | 1.07 (0.89-1.28) | 0.49 | 1.09 (0.89-1.33) | 0.40 |
| Definite stent thrombosis | 1 (1.3) | 33 (0.8) | 63 (0.9) | 31 (0.7) | 1.01 (0.66-1.56) | 0.95 | 0.74 (0.44-1.23) | 0.24 |

Underweight, normal weight, overweight, and obesity are defined as BMI <18.5 kg/m^2^, 18.5-24.9 kg/m^2^, 25.0-29.9 kg/m^2^, and ≥30kg/m^2^, respectively. Because of limited number of underweight patients (N=79, 0.49%), relative risks were evaluated among other 3 groups (normal weight, overweight, and obesity patients). Adjusted covariates in the adjusted model are listed in **Fig. 2** and **Table 3**.

BMI: body mass index; WHO: World Health Organization; MI: myocardial infarction; BARC: Bleeding Academic Research Consortium; HR: hazard ratio; CI: confidence interval.

**Online Table 3. Adjusted hazard ratios according to each BMI value from 18 kg/m^2^ to 40 kg/m^2^**

| BMI | All-cause mortality or new Q-wave MI | BARC type 3 or 5 bleeding |
| --- | --- | --- |
| kg/m^2^ | Adjusted HR (95% CI) | Adjusted HR (95% CI) |
| 18 | 1.71 (1.23-2.38) | 1.69 (1.07-2.65) |
| 19 | 1.61 (1.20-2.15) | 1.58 (1.06-2.36) |
| 20 | 1.51 (1.17-1.94) | 1.49 (1.06-2.10) |
| 21 | 1.41 (1.14-1.75) | 1.40 (1.05-1.88) |
| 22 | 1.33 (1.11-1.58) | 1.32 (1.04-1.67) |
| 23 | 1.24 (1.09-1.42) | 1.24 (1.03-1.49) |
| 24 | 1.17 (1.06-1.29) | 1.17 (1.02-1.33) |
| 25 | 1.10 (1.04-1.17) | 1.10 (1.01-1.19) |
| 26 | 1.04 (1.02-1.07) | 1.04 (1.01-1.08) |
| **27** | **Reference** | **Reference** |
| 28 | 0.97 (0.95-0.99) | 0.97 (0.94-1.00) |
| 29 | 0.96 (0.93-1.00) | 0.95 (0.90-1.00) |
| 30 | 0.96 (0.91-1.01) | 0.94 (0.88-1.01) |
| 31 | 0.97 (0.91-1.04) | 0.94 (0.86-1.04) |
| 32 | 0.99 (0.91-1.09) | 0.95 (0.83-1.08) |
| 33 | 1.02 (0.91-1.14) | 0.96 (0.81-1.14) |
| 34 | 1.05 (0.91-1.21) | 0.98 (0.79-1.21) |
| 35 | 1.08 (0.91-1.28) | 0.99 (0.77-1.28) |
| 36 | 1.11 (0.91-1.35) | 1.01 (0.75-1.35) |
| 37 | 1.14 (0.91-1.44) | 1.02 (0.73-1.44) |
| 38 | 1.18 (0.91-1.52) | 1.04 (0.70-1.52) |
| 39 | 1.21 (0.91-1.61) | 1.05 (0.68-1.62) |
| 40 | 1.25 (0.91-1.71) | 1.07 (0.66-1.71) |

Hazard ratios with 95% CIs correspond to the cubic spline curves shown in **Fig. 2**. The reference value of BMI is 27 kg/m^2^. Adjusted covariates in the adjusted model are listed in **Fig. 2** and **Table 3**.

Abbreviations as in Online Table 2.
